# Pharmacokinetic and Pharmacodynamic Evaluation of Marbofloxacin in Pig against Korean Local Isolates of* Actinobacillus pleuropneumoniae*

**DOI:** 10.1155/2017/2469826

**Published:** 2017-04-06

**Authors:** Md. Akil Hossain, Hae-chul Park, Kyunghun Jeong, Yang ho Jang, Dae Gyun Kim, JeongWoo Kang, Kwang-jick Lee

**Affiliations:** Veterinary Drugs & Biologics Division, Animal and Plant Quarantine Agency (QIA), 177 Hyeoksin 8-ro, Gimcheon-si, Gyeongsangbuk-do 39660, Republic of Korea

## Abstract

The pharmacokinetics of marbofloxacin in pigs after intravenous (i.v.), intramuscular (i.m.), and peroral (p.o.) administration and pharmacokinetic/pharmacodynamic indices of this drug against Korean local isolates of* Actinobacillus pleuropneumoniae* were determined in this study. Marbofloxacin (2.50 mg/kg of body weight) was administered, and blood samples were collected with designated time intervals. Plasma-extracted marbofloxacin was injected into the LC-MS/MS system. The in vitro and ex vivo antibacterial activities of marbofloxacin were evaluated against 20 isolates of* A. pleuropneumoniae*. The mean peak plasma concentrations (*C*_max_) after i.v., i.m., and p.o administration were 2.60 ± 0.10, 2.59 ± 0.12, and 2.34 ± 0.12 *µ*g/mL at 0.25 ± 0.00, 0.44 ± 0.10, and 1.58 ± 0.40 h, respectively. The area under the plasma concentration-time curves (AUC_0–24_) and elimination half-lives were 24.80 ± 0.90, 25.80 ± 1.40, and 23.40 ± 5.00 h·*μ*g/mL and 8.60 ± 0.30, 12.80 ± 1.10, and 8.60 ± 0.00 h, for i.v., i.m., and p.o. administration, correspondingly. The AUC_0–24_/MICs of marbofloxacin after i.v., i.m., and p.o. administration were 253.86 ± 179.91, 264.1 ± 187.16, and 239.53 ± 169.75 h, respectively. The *C*_max_/MIC values were 26.58 ± 18.84, 26.48 ± 18.77, and 23.94 ± 16.97, and T>MICs were 42.80 ± 1.01, 36.40 ± 1.24, and 38.60 ± 1.18 h, after i.v., i.m., and p.o. administration, respectively. Thus, marbofloxacin dosage of 2.50 mg/kg of body weight by i.v., i.m., and p.o. administration with 24 h dosing interval will provide effective treatment for the infection of pig by* A. pleuropneumonia*.

## 1. Introduction

Marbofloxacin is one of the fluoroquinolones that were widely used in veterinary medicine and exhibits concentration-dependent bactericidal activity [1, 2]. This antibiotic has a wide spectrum of activity, mainly against gram-negative pathogens, some gram-positive pathogens, and* Mycoplasma spp*. [2]. Its properties of rapid absorption, good distribution, and broad spectrum against most of the swine respiratory pathogens, such as* Haemophilus parasuis* and* Actinobacillus pleuropneumoniae*, make it a good candidate to deal with a respiratory outbreak caused by any of these pathogens [3].* A. pleuropneumoniae* is the causative agent of porcine pleuropneumonia, a worldwide disease with occasional clinical outbreaks that can have a severe economic impact [4]. Attempts to control the disease have been made by vaccination, treatment with antibiotics, and the establishment of herds free of the infection. Pigs can become asymptomatic carriers of the organism in their tonsils for long period of time [5, 6], thereby exposing susceptible animals and maintaining the disease in the herd. Moreover, pigs can carry* A. pleuropneumoniae* in their tonsils for several months without seroconverting [7]. Eradication of* A. pleuropneumoniae* from pig herds has been made with different antibiotics [4–7]. But,* A. pleuropneumoniae* is gaining resistance against all kinds of antibacterial agents including marbofloxacin [8].

It was reported that the resistance rates of fluoroquinolones were increasing in several European countries as well as in Korea, Taiwan, and Japan [8]. International organizations, such as the World Health Organization (WHO), the Food and Agriculture Organization of the United Nations (FAO), and the World Organization for Animal Health (OIE), as well as regulating authorities, have expressed their concern with the development of resistance in microorganisms that are pathogenic both for humans and animals particularly to some antimicrobial classes, including fluoroquinolones [9]. Currently, no fluoroquinolones are approved for use in poultry in the US [10]. The drugs are also prohibited in chicken farms in Australia, Finland, and Denmark [11]. Conversely, the law (number: 2014-1170) published in October 2014 in France targeted reducing the use of fluoroquinolones and third- and fourth-generation cephalosporins by 25% before December 2016, but not restricted completely [12]. Yet unpublished figures compiled by the British Poultry Council (BPC), which represent around 90% of the UK industries, reveal its members have increased their use of these drugs, using 1.126 tonnes of fluoroquinolones in 2014 compared with 0.71 tonnes in the previous year, which indicates that these antimicrobials are still in use in many countries [11].

However, there are recent concerns about the emergence of quinolone-resistant bacterial strains and the impact of improper use of these drugs on human and animal; fluoroquinolones are still an important antimicrobial medicine. Thus, there is an important need to use fluoroquinolones with caution to preserve their effectiveness for many years. In veterinary medicine, it is essential to reserve these drugs for cases requiring a powerful antibiotic and to prescribe and/or administer them only under a good clinical assessment and with appropriate regimens [12]. Because, the selection of improper dose and dose intervals are accelerating the resistance against these drugs [13–16]. The study of pharmacokinetics and pharmacodynamics and their integration is the major tool to determine the dosage regimens appropriately. The determination of pharmacokinetic parameters and their interspecies differences helps to minimize dosage errors. The pharmacokinetics of marbofloxacin have been investigated in different animals and it demonstrated an almost 100% bioavailability, higher concentration in plasma and peripheral tissues in goats [17], cows [18], cats [19], sheep [20], dogs [21], and pigs [22, 23]. Although, some studies have been published on the pharmacokinetics of marbofloxacin in animals including pigs [3, 18–22, 24, 25], yet the pharmacokinetic data of marbofloxacin in pig tissues and plasma is not sufficient enough to predict the efficacy of this drug precisely. Furthermore, the integration of pharmacokinetic (PK) data with pharmacodynamic (PD) data helps to establish the PK/PD indices (AUC/MIC, *C*_max_/MIC, *T* > MIC, AUC > MIC, etc.,) which are fundamental to predict efficacy and minimize resistance development [26, 27].

Since there are contradicting values in PK/PD indices which correlate with prevention of resistant mutant selection and efficacy [28, 29], determining PK/PD indices specific to a particular pathogen and antimicrobial agent has received great attention. The integration of PK data with the time course activity (ex vivo) of marbofloxacin against* A. pleuropneumoniae* has not been studied in pigs. Thus, it was intended firstly to develop and validate a sensitive and reliable LC-MS/MS method for attaining the ultimate goal while the prime objective of this study was to characterize the pharmacokinetics of marbofloxacin following i.v., i.m., and p.o. administration at a dose of 2.50 mg/kg of body weight in pigs and to explore in vivo and ex vivo PK/PD indices using Korean local pathogenic strains of* A. pleuropneumoniae* as a model bacterium.

## 2. Materials and Methods

### 2.1. Chemical Reagents and Media

Marbofloxacin (Marbocyl 10% solution) was purchased from Vetoquinol Ltd. (Lure, France). Marbofloxacin reference standard was purchased from United States Pharmacopeial Convention (Rockville, MD, USA). Mueller-Hinton broth (MHB) and chocolate agar media were obtained from Difco Laboratories (Detroit, MI, USA), and veterinary fastidious medium (VFM) was from Thermo Fisher Scientific (Waltham, MA, USA). Other reagents and chemicals were of analytical grade. Purified water was prepared using the Milli-Q water purification system from Millipore, Inc. (Bedford, MA, USA).

### 2.2. Animal Experimental Procedure

The study was carried out in 12-week-old castrated cross-bred (Duroc × Landrace × Yorkshire) healthy male pigs at Animal and Plant Quarantine Agency, Gimcheon-si, South Korea. Pigs with body weight of about 30 kg were purchased from the Sunjin CU farm (Icheon, Gyeonggi-do, South Korea). All animals were kept in a self-contained animal unit. Commercial antibiotic-free feed and fresh water were provided ad libitum throughout the experimental period. The pigs were fasted for overnight (~12 h) and randomly divided into 3 groups, where 4 pigs were assigned in each group. Marbofloxacin (Marbocyl 10% Solution, Vetoquinol Ltd., Lure, France) at a dose of 2.5 mg/kg of body weight was administered to different groups of animals through i.v., i.m., and p.o. routes for the determination of the basic pharmacokinetic parameters. All animal experimental procedures were approved by the Institutional Animal Care and Use Committee of Animal and Plant Quarantine Agency, Gimcheon-si, South Korea (approval number: 2014-2190).

### 2.3. Blood Collection and Sample Preparation

The blood samples (5 mL) were taken in heparinized Vacutainer tubes by puncturing of jugular vein at 0 min, 15 min, 30 min, 45 min, 1 h, 1.5 h, 2 h, 3 h, 6 h, 9 h, 12 h, 18 h, 24 h, 30 h, 36 h, 48 h, 60 h, 72 h, and 120 h from all of these animals. The blood samples were centrifuged at 2000 ×g for 10 min at 4°C to obtain the plasma. The plasma samples (500 *μ*L) were mixed with 1.5 mL of 0.1% formic acid in acetonitrile to precipitate plasma proteins. After shaking for 20 min, the mixtures were centrifuged at 5000 ×g for 30 min. The solvents of the supernatants were evaporated under nitrogen flow at 50°C to make the volume about 500 *μ*L and then stored in −70°C refrigerator.

### 2.4. LC-MS/MS Analysis

Liquid chromatography-tandem mass spectrometry (LC-MS/MS) with gradient elution through YMC C_18_ (3.0 × 100 mm, 3 *μ*m) column was utilized to determine the content of marbofloxacin in plasma by analyzing 5 *μ*L aliquot of each samples. The mobile phase was a mixture of (A) 0.1% formic acid in distilled water and (B) 0.1% formic acid in acetonitrile, where the ratios of “A” and “B” were different and maintained in a gradient flow. The initial composition of mobile phase was 90% of “A” and 10% of “B” which was linearly changed to 100% of “B” from 0.1 to 3 min and maintained this ratio up to 4.9 min. At 5 min, the ratio was directly returned to its base composition (10% B) and maintained this composition up to the end of the acquisition. The flow rate was 0.6 mL/min, and the injection volume was 5 *μ*L. Tandem mass spectrometry with electrospray ionization was used and maintained in positive mode. The mass to charge ratios (*m/z*) of precursor ions, quantification ions and confirmation ions, and the collision energies (CE) of marbofloxacin are listed in Table 1. The method was optimized and validated prior to applying for PK analysis.

### 2.5. Validation of the Analytical Method

Specificity was determined from three blanks and pooled plasma samples which were analyzed to note the absence of interferences in the elution position of marbofloxacin. Stock solution of marbofloxacin (1 mg/mL) was prepared by dissolving a pure reference standard of marbofloxacin in aqueous solution of 0.1% formic acid to stabilize the pH for establishing the linearity. Further dilutions of the stock solution were prepared, and the drug solutions of different concentrations were spiked into blank plasma to produce calibration curves. Linearity was determined by a series of three injections of six plasma samples spiked with (5, 10, 50, 100, 500, and 1000 ng/mL) marbofloxacin. Pooled plasma samples were spiked with known concentrations (10 and 100 ng/mL) of marbofloxacin and deproteinated with acetonitrile for the determination of accuracy and recovery. After extraction of the analyte from the matrix and injection to the analytical instrument, the recovery was determined by comparing the resulting peak response with those of other samples to which the same corresponding concentrations of the drug had been added just before the injection.

Repeatability and reproducibility were studied on six injections from spiked plasma samples of three different concentrations. The detection limit and quantitation limit were determined from the calibration curve by analyzing marbofloxacin spiked samples. The limit of detection (LOD) was calculated from the standard deviation of responses and the slope obtained from the calibration curve as stated by the following equation: LOD = (3.3 × SD)/slope [30, 31]. The limit of quantitation (LOQ) was calculated from the standard deviation (SD) of responses and the slope associated with the calibration curve, according to the following equation: LOQ = (10 × SD)/slope [30, 31].

### 2.6. Pharmacokinetic Study

The concentrations of marbofloxacin in plasma samples of different time points were analyzed by LC-MS/MS. The pharmacokinetic parameters of marbofloxacin were analyzed by WinNonlin 6.1 software (Pharsight Corporation, Mountain View, CA, USA). The area under the curve (AUC), peak times (*T*_max_), peak plasma concentrations (*C*_max_), elimination half-life (*T*_1/2_), and absolute bioavailability (*F*) were calculated for pharmacokinetic determination using a noncompartmental analysis. The absolute bioavailability for i.m. administration was determined with the formula *F* = (AUC_im_/AUC_iv_) × 100%. Similarly, absolute bioavailability was determined by *F* = (AUC_po_/AUC_iv_) × 100%, in the case of p.o. administration. AUC_iv_, AUC_im_, and AUC_po_ are obtained after i.v., i.m., and p.o. administration, respectively. Pharmacokinetic parameters were calculated for each individual animal and are presented as arithmetic means ± SD.

### 2.7. Ex Vivo Experiment

Plasma samples were collected from pigs after 0, 1/4, 3/4, 2, 9, and 24 h of marbofloxacin administration via i.v., i.m., and p.o., which were used for ex vivo experiment. Controls were prepared from plasma samples collected from those pigs which did not receive any drug. About 170 *μ*L of VFM (veterinary fastidious medium) was added to each well of a 96-well microtitre plate. The bacterial cultures (20 *μ*L) of* A. pleuropneumoniae* (*n* = 20) from the stationary-phase of growth were individually added to 10 *μ*L of plasma and mixed with the 170 *μ*L of VFM in each well to give a final concentration of approximately 10^6^ CFU/mL. One hundred microlitre of sample was collected at each time point, serially diluted, and spread over chocolate agar media. The bacteria in plates were further incubated at 35°C with 5% CO_2_ for 24 h. After incubation, bacteria colonies were manually counted.

### 2.8. Pharmacodynamic Study

The minimum inhibitory concentration (MIC) and minimum bactericidal concentration (MBC) of marbofloxacin against 20 isolates of* A. pleuropneumoniae* were determined by using Mueller-Hinton broth (MHB) and Mueller-Hinton Agar (MHA) according to the standard broth microdilution method as described in Clinical and Laboratory Standards Institute (CLSI) guideline [32]. Marbofloxacin was serially diluted twofold horizontally from first well to tenth well in 96-well plates where the final concentrations would be from 32 to 0.00391 *μ*g/mL after inoculation of bacterial culture. Bacterial culture from midlogarithmic phase was diluted and 100 *μ*L of the diluted bacterial suspension was added to serially diluted-drug solutions in 96-well plates where the final inoculum density would be approximately 5 × 10^5^ CFU/mL. The bacteria in presence of drug substances in 96-well plates were incubated at 35°C for 18 h. After incubation, the lowest concentration that inhibited visible growth of microorganism was considered as MIC. Cultures (20 *μ*L) from all microwells in 96-well plates that showed no visible growth were spotted on Tryptic Soy Agar (TSA) plates and incubated for 24 h at 35°C. The lowest concentration that completely inhibited growth on agar plate was considered as minimum bactericidal concentration (MBC). All experimentations were performed in triplicate.

### 2.9. Data Analysis

Data are presented as mean ± standard deviation of three replicate assays. Analysis of variance (ANOVA) and *F*-test were performed and *P* values of less than 0.05 were considered to be statistically significant.

## 3. Results

### 3.1. Validation of Analytical Method

**Figure 1:**
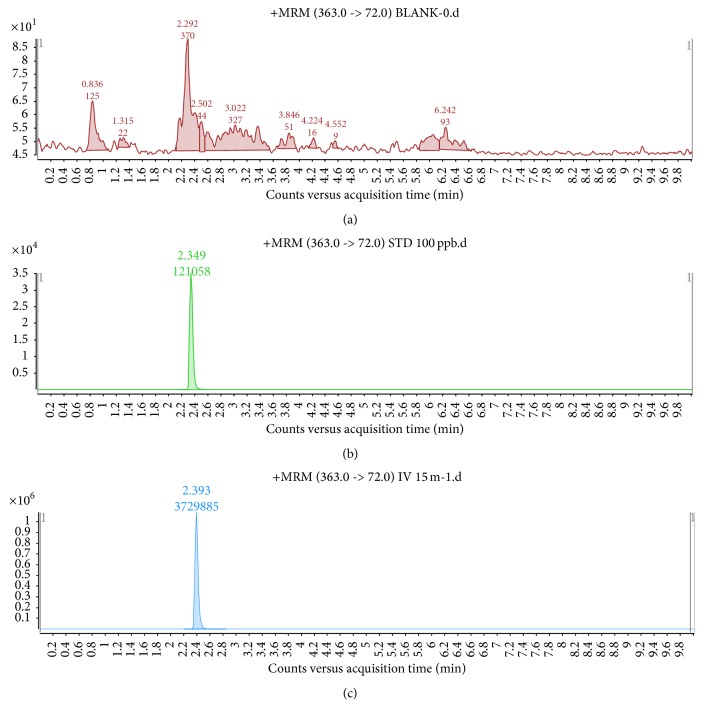
Mass chromatograms of (a) blank plasma, (b) standard marbofloxacin solution, and (c) marbofloxacin spiked plasma samples. (This figure is obtained from the LC-MS/MS system and it is not possible to edit).

The method validation was performed in the sample matrix. Good linearity (*R*^2^ > 0.999) was observed, and the quantified average recoveries of marbofloxacin were 87−92% at the level of 10 to 100 ng/mL. The within-run precision (percent coefficient of variation, % CV) for the described method was less than 10% over the range of concentrations studied. The limits of detection (LOD) and the limit of quantification (LOQ) were 2 and 5 ng/mL, respectively. The validation data of the analytical method is presented in Table 2, and the representative LC-MS/MS chromatograms of blank serum and standard and spiked sample solutions are shown in Figure 1.

### 3.2. Pharmacokinetics of Marbofloxacin in Plasma

**Figure 2:**
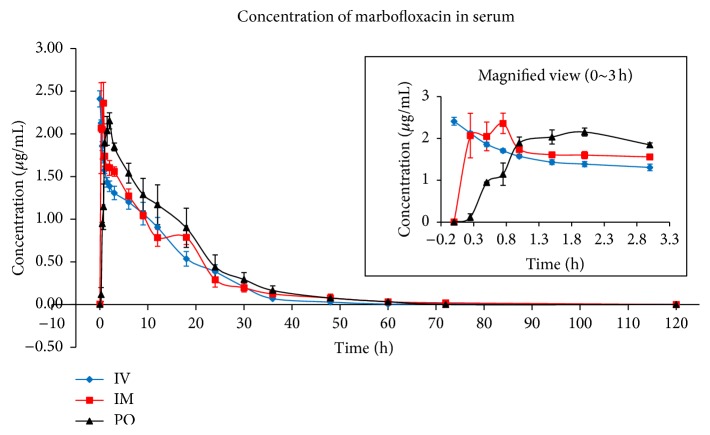
The plasma concentrations (mean ± SD) of marbofloxacin versus time after intravenous, intramuscular, and peroral administration to pigs.

The concentrations of marbofloxacin in plasma at different time intervals after i.v., i.m., and p.o. administration are presented in Figure 2. Pharmacokinetic parameters of marbofloxacin after i.v., i.m., and p.o. administrations are shown in Table 3. The peak plasma concentrations (*C*_max_) of marbofloxacin were 2.60 ± 0.10 *μ*g/mL, 2.59 ± 0.12 *μ*g/mL, and 2.34 ± 0.12 *μ*g/mL for i.v., i.m., and p.o. administration, respectively. The areas under the plasma concentration-time curves (AUC_0–24_) were 24.80 ± 0.90, 25.80 ± 1.40, and 23.40 ± 5.00 h·*μ*g/mL for i.v., i.m., and p.o. administration, correspondingly. The elimination half-life (*T*_1/2_) values were 8.60 ± 0.30, 12.80 ± 1.10, and 8.60 ± 0.00 h, correspondingly in i.v., i.m., and p.o. administration. Absolute percentages of bioavailability (*F*) of the marbofloxacin in pig were 104.60 ± 5.70% and 94.35 ± 8.90%, respectively, in intramuscular and peroral routes compared to intravenous route.

### 3.3. Ex Vivo Antibacterial Effect

**Figure 3:**
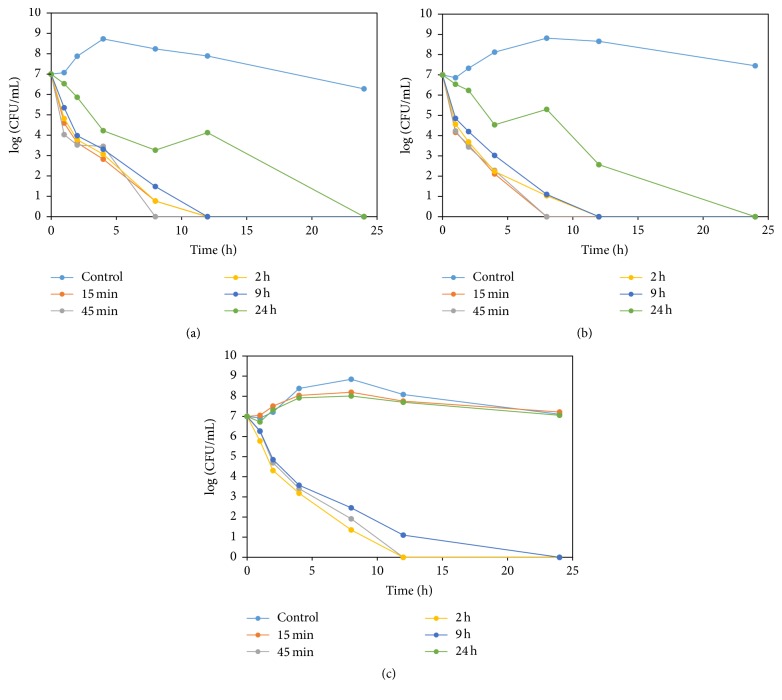
The results of ex vivo antibacterial effect of marbofloxacin using various routes of administration against 20 isolates of* Actinobacillus pleuropneumoniae*. (a) Intravenous, (b) intramuscular, and (c) peroral administration.

The time-dependent antibacterial effects of marbofloxacin against* A. pleuropneumoniae* in ex vivo condition were determined in plasma samples which were collected at 0, 1/4, 3/4, 2, 9, and 24 h after i.v., i.m., and p.o. administration of marbofloxacin in pigs (Figure 3). The addition of plasma samples which were collected at 1/4 h to 9 h after the i.v. administration of marbofloxacin showed bactericidal effect within 12 h of incubation. The supplementation of plasma that was collected at 24 h of marbofloxacin administration showed a significant reduction of bacterial growth (log CFU/mL) at different time points before 24 h and completely eliminated the bacteria at 24 h of incubation. The incubation of bacteria in presence of plasma samples which were collected after i.m. administration also demonstrated similar trend of bacterial elimination. When* A. pleuropneumoniae* isolates were incubated with the addition of plasma which were collected at 3/4, 2, and 9 h of administration through p.o. route, they also displayed bactericidal effect. However, there were no noticeable variations in bacterial (log CFU/mL) growth, when the plasma samples were collected at 1/4 and 24 h of p.o. administration and the bacteria were incubated in presence of those plasma samples.

### 3.4. In Vitro MICs and MBCs of Marbofloxacin

The MICs and MBCs of marbofloxacin against 20 isolates of* A. pleuropneumonia* were determined. The MICs of marbofloxacin against these isolates were ranged from 0.03152 to 1.00 *μ*g/mL. The MBCs of this antimicrobial drug against those strains of* A. pleuropneumoniae* were from 0.0625 to 8.00 *μ*g/mL. The ratio of MIC and MBC was 1 for 6 strains out of 20. The MBC values were 2- to 16-fold higher than MIC values against most of the strains. The MIC, MBC, and the ratio of MIC and MBC for each strain are presented in Table 4.

### 3.5. PK/PD Integration of Marbofloxacin in Serum

The in vitro MIC and MBC data were integrated with in vivo PK data to determine the PK/PD indices such as AUC_0–24_/MIC, AUC_0–24_/MBC, *C*_max_/MIC, *C*_max_/MBC,* T* > MIC, and AUC_0–24_ > MIC, which are presented in Table 5. The AUC_0–24_/MIC and AUC_0–24_/MBC ratios of marbofloxacin against* A. pleuropneumoniae* after i.v., i.m., and p.o. administration were 253.86 ± 179.91, 264.1 ± 187.16, and 239.53 ± 169.75 h and 144.93 ± 143.35, 150.77 ± 149.13, and 136.74 ± 135.26 h, respectively. The time where the plasma concentration exceeds MIC (*T* > MIC) after i.v., i.m., and p.o. administration was 42.80 ± 1.010, 36.40 ± 1.24, and 38.60 ± 1.18 h, respectively. The AUC_0–24_/MIC, AUC_0–24_/MBC, *C*_max_/MIC, and *C*_max_/MBC values attained from the application of marbofloxacin through p.o. route are significantly lower than i.v. and i.m. administration values. On the contrary, the AUC_0–24_ > MIC value with the administration of marbofloxacin through p.o. route is significantly higher than the values obtained from i.v. and i.m. administration. There were no noticeable variations in PK/PD indices of plasma samples collected after i.v. and i.m. administration to pigs.

## 4. Discussion

In the current study, a sensitive and reliable LC-MS/MS method is developed for the rapid detection of marbofloxacin in plasma. This validated method was applied in determining pharmacokinetics and pharmacokinetic-pharmacodynamic indices of marbofloxacin after i.v., i.m., and p.o. administration in pigs. The pharmacokinetic study of marbofloxacin in this investigation is in accordance with previous studies in horses [33], beagle dogs [34], and hanwoo, Korean native cattle [35]. The current study demonstrated a favorable pharmacokinetic profile of marbofloxacin in pigs in terms of the rapid absorption of the drug from the tissues to the systemic circulation, prolonged duration of action as evident by the long terminal half-life, and excellent relative bioavailability. The probability of clinical success was evaluated through the use of the AUC_0–24_/MIC and *C*_max_/MIC index.

We developed the LC-MS/MS method in the current study in order to identify and quantify marbofloxacin simultaneously from plasma. In LC-MS/MS chromatogram (Figure 1), peak of pure marbofloxacin was observed at about 2.4 minutes. When the plasma-spiked marbofloxacin solution was injected into the LC-MS/MS systems, the retention time and the mass to charge value (*m*/*z*) of the spiked marbofloxacin were found to be similar as it was obtained in the solution of pure compound (Figure 1). A fast, simple, and efficient extraction procedure is one of the essential parts of the quantification method in the present study. In this study, the totality of the plasma supernatant was dried by nitrogen evaporation to increase the signal of marbofloxacin. The major advantages of the present method were a shorter extraction time and that there is no need of a derivatization step. Therefore, the procedure described here enables the direct extraction of marbofloxacin without complementary purification steps.

The precision or coefficient of variation (%) of this assay method was within the limits for the tested concentrations according to the guidelines for analytical method development and validation [36, 37], which indicates that the assay method is validated depending on the precision. The acceptance criterion for linearity is that the correlation coefficient (*r*^2^) should not be <0.990 for the least squares method of the analysis of the line [24]. The correlation coefficient of marbofloxacin was 0.9999 (Table 2). This result demonstrates the linearity of this method over a wide dynamic range.

The recovery percentage of this drug compound (Table 2) may be considered as slightly lower than expected, which could be attributable to the interference of the sample matrix or the slowness of extraction of this compound from the sample matrix. Moreover, the slightly lower recovery is justified in this case, as the expected recovery is dependent on the percentage of analyte in the matrix [37], indicating that the analytical method is validated. Specificity of the analytical method ensures that the signal measured comes from the desired compound and there is no interference from diluents, extract materials, and mobile phase. The data obtained in the validation study proved that the proposed method is validated and can be used for the determination and quantification of marbofloxacin in plasma.

The mean (±SD) plasma concentration versus time curves of marbofloxacin at a dose of 2.5 mg/kg of body weight in pigs following i.v., i.m., and p.o. administration is shown in Figure 2. The administered dose is recommended to be daily in pigs [22]. Marbofloxacin was measurable in pig plasma from just after the administration to 72 h after i.v. and i.m. administration routes whereas, the drug substance was quantified from 15 min to 120 h after p.o. administration. Excluding the first part of the curve (0–0.75 h), the mean plasma concentrations of the administered three dosages were almost identical.

The longer elimination half-life of marbofloxacin observed in this study reflects the advantage of this drug in maintaining effective concentration in the body thereby allowing longer time for drug-pathogen interaction. The elimination half-life of marbofloxacin in pigs after i.v. administration was comparable with values in pigs, rabbits, and goats [17, 23, 26] and longer than the value in sheep (3.96 h) [20]. After i.m. administration, the elimination half-life of marbofloxacin in the present study (12.8 h) was shorter than that obtained in pigs (17.3 h) [23], much longer than those obtained in goats (6.77 h) [17], horses (5.74 h) [33], calves (4.7 h) [38], and camels (7.98 h) [39], and also apparently longer than that obtained after i.v. administration in this study. This difference is probably the result of continued absorption of marbofloxacin from the i.m. injection site during the elimination phase, thereby prolonging the* t*_1/2_ of the drug. The elimination half-life of marbofloxacin in our study following single p.o. administration in pig was estimated to be 8.6 h, shorter than that in sea turtles (13.33 h) [40] but similar to that in chickens (8.69 h) [25]. The elimination half-life of this drug after p.o. administration in pig was largely higher in a previous investigation (23.14 h) [25] than the value observed in the current study.

The *T*_max_ of marbofloxacin following single p.o. administration was estimated to be 1.58 h, which is similar to that obtained in chickens (1.48 h) [23]. The *T*_max_ after i.m. administration in hanwoo cows (0.95 h) [35] was higher and in beagle dogs (0.47 h) [34] and sea turtles (0.65 h) [40] was comparable with the *T*_max_ value in pigs (0.44 h) in our study. *T*_max_ in sea turtles (0.30 h) [40] is similar to the value (0.25 h) in our study in pigs after i.v. administration. Marbofloxacin was rapidly absorbed with a *C*_max_ of 2.59 ± 0.12 *μ*g/mL achieved at 0.44 ± 0.10 h after i.m. administration. The *C*_max_ values of marbofloxacin achieved after i.m. and p.o. administration were higher than the MIC break point of fluoroquinolones recommended against most susceptible bacterial species [32]. These values were higher than the values reported in goats (1.87 *μ*g/mL) [17], rabbits (2.04 *μ*g/mL) [20], and sheep (0.80 *μ*g/mL) [25]. Even, the *C*_max_ value of marbofloxacin in this study was higher than the previously reported values in pigs (1.81 *μ*g/mL in i.m. and 1.03 *μ*g/mL in p.o.) [23]. The relative bioavailability of marbofloxacin was calculated to be 104.60 ± 5.70% after i.m. administration, while high bioavailability has also been reported in rabbits (123.30%) [40] and in goats (100.74%) [17]. The relative bioavailability obtained for marbofloxacin after p.o. application was 94.35 ± 8.90%, which is in between the bioavailabilities (91.50 ± 13.70% and 107.90 ± 13.40%) reported previously [22, 23]. In this study, higher intramuscular marbofloxacin bioavailability (more than 100%) may be because of the prolonged elimination half-lives after i.m. administration that could have induced higher AUC values.

The ex vivo killing study indicates concentration-dependent antibacterial effect of marbofloxacin; increasing the drug concentration led to more-rapid killing of all tested bacterial strains. The values of ex vivo antibacterial effects are rational according to the in vitro MIC and MBC data, and serum concentrations of marbofloxacin at different time points. Roughly, the serum concentrations of marbofloxacin were above the in vitro MBC concentrations of most of the strains at time (0−9) h, (0.25−9) h and (0.5−12) h for i.v., i.m. and p.o. administrations, respectively. Moreover, the elimination half-lives of marbofloxacin from all of these dosage forms in pigs are longer, which also justifies the ex vivo antibacterial effect of marbofloxacin. The integration of PK data with the PD data presents a better approach to dose titration studies for selecting rational dosage regimens in veterinary medicine [27]. Furthermore, the PK/PD indices of the same drug against different pathogens also vary. So, it is of great importance to study the PK/PD indices of fluoroquinolones against individual pathogen [41]. In the current study, the PK data obtained from a single i.v., i.m., and p.o. administration of marbofloxacin in pigs was integrated with PD (ex vivo) data using* A. pleuropneumoniae* as a model organism. Large AUC_0–24 h_/MIC and *C*_max_/MIC ratios were obtained for* A. pleuropneumonia* isolates following i.v., i.m., and p.o. administration of marbofloxacin in pigs (Table 5). PK/PD indices, such as *C*_max_/MIC and AUC_0–24 h_/MIC values of the target organism, have been used to predict the clinical efficacy of antibacterial agents. Another important PK/PD parameter to describe drug efficacy is the time during which the drug concentration exceeds the MIC (*T* > MIC) [42]. It is generally recommended that* T* > MIC should be at least 50% of the dosage interval to ensure an optimal bactericidal effect [42, 43]. During the present study, to optimize the marbofloxacin dosage regimen, we also calculated the* T* > MIC at a dose of 2.50 mg/kg of body weight in pigs after i.v., i.m., and p.o. administration.

The values of PK/PD parameters (AUC/MIC, *C*_max_/MIC, and* T* > MIC) obtained in this study were compared with the PK/PD values in previous studies. The AUC/MIC and* T* > MIC of marbofloxacin in serum against* Mannheimia haemolytica* after i.v. and i.m. administration to calf at a dosage of 2.0 mg/kg were 249.75 ± 25.87 and 252.67 ± 9.16 h and 22.69 ± 1.69 and 22.68 ± 0.79 h, respectively. The ratio of *C*_max_ and MIC in the same situation after i.m. administration was 37.60 ± 1.87 [1], where the AUC/MIC values in calf are similar as obtained in the current study. In sheep, the AUC/MIC in serum after i.v. and i.m. dosing against* Mannheimia haemolytica* were 120.2 and 135.5 h, respectively. After i.m. administration* T* > MIC was 10.5 h, and *C*_max_/MIC was 21.1 which is comparable with the *C*_max_/MIC value of this study [20]. The AUC/MIC, *C*_max_/MIC, and* T* > MIC of marbofloxacin against* Escherichia coli* after oral administration (2 mg/kg of body weight) in turkeys were 73.69 ± 25.54 h, 5.35 ± 2.31, and 10.9 h, which are about 4 times lower than the values of corresponding parameters in the present study [44]. AUC_24 h_/MIC and* T* > MIC of marbofloxacin against* Staphylococcus pseudintermedius* after i.v. and i.m administration (2 mg/kg body weight) in beagle dogs were 67.76 ± 1.23 h and 91.18 ± 2.61 h and 9.83 ± 1.72 and 15.50 ± 6.68, respectively, and the *C*_max_/MIC after i.m. administration in the same situation was 13.04 ± 1.03. All the values of PK/PD parameters in beagle dogs are about half the values obtained in the current study [34]. The AUC_0–24 h_/MIC values 253.86, 264.10, and 239.53 h after i.v., i.m., and p.o. administration, respectively, are likely to provide a good antibacterial outcome. So that, the 2.50 mg/kg dosage may be regarded as appropriate for the strains of* A. pleuropneumonia* used in this study [44], as the AUC_0–24 h_/MIC ratio of >125 h and a *C*_max_/MIC ratio of >10 are generally considered the best indicators of activity for agents with concentration-dependent killing and were usually used as a threshold for successful therapeutic outcome of fluoroquinolones against gram-negative bacteria [45]. Nevertheless, these thresholds may be different for some fluoroquinolones. The greatest influence for the differences was the immune status of the animal. The plasma concentrations were also greater than the MICs of the tested drug. It can be assumed that marbofloxacin concentration in the site of action was at least very similar to that observed in plasma due to the high bioavailability, low protein binding, and tissue distribution reported for fluoroquinolones [46].

## 5. Conclusions

The current study demonstrated promising pharmacokinetic profiles of i.v., i.m., and p.o. formulations of marbofloxacin in pigs. Further, the current study established the correlation between the plasma concentration of marbofloxacin and its in vitro activity against an important bacterial pathogen,* A. pleuropneumonia*. It was also revealed in this study that the marbofloxacin was completely absorbed and slowly eliminated after single i.v., i.m., and p.o. administration in healthy pigs. The mean marbofloxacin plasma concentrations at 24 h after i.v., i.m., and p.o. administration at a dose of 2.50 mg/kg were all higher than 0.25 *μ*g/mL, which are above or very close to the MIC_90_ against most major pathogenic bacteria [47, 48]. So, a marbofloxacin dosage of 2.50 mg/kg of body weight by i.v., i.m., or p.o. administration to pigs with 24 h dosing interval will provide effective treatment for the infection of pig by* A. pleuropneumonia*. Additional studies may also be necessary to confirm the penetration of marbofloxacin in diseased tissues, so that its potential use in clinical situations could be assessed.

## Figures and Tables

**Table 1:** Mass to charge ratios (*m/z*), collision energies (CE) of precursor ions, quantification ions, and confirmation ions.

Substance	Precursor ion *m/z*	Quantification ions *m/z* (CE, eV)	Confirmation ions *m/z* (CE, eV)
Marbofloxacin	363	72 (110, 20)	320 (110, 15)

**Table 2:** Validation parameters of marbofloxacin by LC-MS/MS.

Analyte	RT (min)	Correlation coefficient (*r*^2^)	Average recovery (%)		Coefficient of variation (%)		LOD (ng/mL)	LOQ (ng/mL)
			10 ng/mL	100 ng/mL	10 ng/mL	100 ng/mL		
Marbofloxacin	2.4	0.9999	92	87	4	5	2	5

**Table 3:** Pharmacokinetic parameters of marbofloxacin following intravenous, intramuscular, and peroral administration using WinNonlin (*n* = 4, mean ± SD).

Parameters	Unit	i.v.	i.m.	p.o.
*T*_1/2_	h	8.60 ± 0.30	^*∗*^12.8 ± 1.10	8.60 ± 0.00
*T*_max_	h	0.25 ± 0.00	0.44 ± 0.10	^*∗*^1.58 ± 0.40
*C*_max_	*µ*g/mL	2.60 ± 0.10	2.59 ± 0.12	2.34 ± 0.12
AUC_0–24_	h·*µ*g/mL	24.80 ± 0.90	25.8 ± 1.40	23.40 ± 5.00
*F*	%	—	104.60 ± 5.70	94.35 ± 8.90

i.v.: intravenous, i.m.: intramuscular, p.o.: peroral, and *T*_1/2_: elimination half-life. *T*_max_: time of maximum concentration; *C*_max_: maximum concentration after administration; AUC_0–24_: area under the serum concentration-time curve from time zero to 24 h; and *F*: bioavailability. ^*∗*^Significantly different among groups.

**Table 4:** The pharmacodynamic parameters of marbofloxacin against 20 isolates of *Actinobacillus pleuropneumonia*.

Strain number	Concentration range (*μ*g/mL)	MIC (*μ*g/mL)	MBC (*μ*g/mL)	MBC/MIC
1	32–0.00391	0.0625	0.0625	1
2	32–0.00391	0.0625	0.25	4
3	32–0.00391	0.125	0.125	1
4	32–0.00391	0.125	0.125	1
5	32–0.00391	0.0625	0.5	8
6	32–0.00391	0.125	0.25	2
7	32–0.00391	0.125	1	8
8	32–0.00391	0.125	0.25	2
9	32–0.00391	0.125	0.25	2
10	32–0.00391	0.0625	0.0625	1
11	32–0.00391	0.5	2	4
12	32–0.00391	0.5	2	4
13	32–0.00391	0.0625	0.25	4
14	32–0.00391	0.125	0.125	1
15	32–0.00391	0.25	2	8
16	32–0.00391	0.125	0.25	2
17	32–0.00391	0.0313	0.0625	2
18	32–0.00391	0.0625	0.0625	1
19	32–0.00391	1	8	8
20	32–0.00391	0.25	4	16

**Table 5:** Pharmacokinetic/pharmacodynamic integration of marbofloxacin in pig after administration through intravenous, intramuscular, and peroral routes.

Parameters	Units	i.v.	i.m.	p.o.
AUC_0–24_/MIC	h	253.86 ± 179.91	264.1 ± 187.16	239.53 ± 169.75^*∗*^
*C*_max_/MIC	—	26.58 ± 18.84	26.48 ± 18.77	23.94 ± 16.97^*∗*^
AUC_0–24_/MBC	h	144.93 ± 143.35	150.77 ± 149.13	136.74 ± 135.26^*∗*^
*C*_max_/MBC	—	15.18 ± 15.01	15.12 ± 14.95	13.67 ± 13.52^*∗*^
*T* > MIC	—	42.80 ± 1.010	36.40 ± 1.24	38.60 ± 1.18
AUC > MIC	—	20.90 ± 0.90	20.40 ± 1.40	26.50 ± 5.00^*∗*^

i.v.: intravenous, i.m.: intramuscular, and p.o.: peroral. ^*∗*^Significantly different among groups.
